# Phosphatidylserine-targeting antibodies augment the anti-tumorigenic activity of anti-PD-1 therapy by enhancing immune activation and downregulating pro-oncogenic factors induced by T-cell checkpoint inhibition in murine triple-negative breast cancers

**DOI:** 10.1186/s13058-016-0708-2

**Published:** 2016-05-11

**Authors:** Michael J. Gray, Jian Gong, Michaela M. S. Hatch, Van Nguyen, Christopher C. W. Hughes, Jeff T. Hutchins, Bruce D. Freimark

**Affiliations:** 1 Department of Preclinical Research, Peregrine Pharmaceuticals, Inc., Tustin, CA USA; 2 Department of Molecular Biology and Biochemistry, University of California, Irvine, CA USA

**Keywords:** Phosphatidylserine, Checkpoint inhibitor, Combination immunotherapy, Breast cancer, Triple-negative breast cancer

## Abstract

**Background:**

The purpose of this study was to investigate the potential of antibody-directed immunotherapy targeting the aminophospholipid phosphatidylserine, which promotes immunosuppression when exposed in the tumor microenvironment, alone and in combination with antibody treatment towards the T-cell checkpoint inhibitor PD-1 in breast carcinomas, including triple-negative breast cancers.

**Methods:**

Immune-competent mice bearing syngeneic EMT-6 or E0771 tumors were subjected to treatments comprising of a phosphatidylserine-targeting and an anti-PD-1 antibody either as single or combinational treatments. Anti-tumor effects were determined by tumor growth inhibition and changes in overall survival accompanying each treatment. The generation of a tumor-specific immune response in animals undergoing complete tumor regression was assessed by secondary tumor cell challenge and splenocyte-produced IFNγ in the presence or absence of irradiated tumor cells. Changes in the presence of tumor-infiltrating lymphocytes were assessed by flow cytometry, while mRNA-based immune profiling was determined using NanoString PanCancer Immune Profiling Panel analysis.

**Results:**

Treatment by a phosphatidylserine-targeting antibody inhibits in-vivo growth and significantly enhances the anti-tumor activity of antibody-mediated PD-1 therapy, including providing a distinct survival advantage over treatment by either single agent. Animals in which complete tumor regression occurred with combination treatments were resistant to secondary tumor challenge and presented heightened expression levels of splenocyte-produced IFNγ. Combinational treatment by a phosphatidylserine-targeting antibody with anti-PD-1 therapy increased the number of tumor-infiltrating lymphocytes more than that observed with single-arm therapies. Finally, immunoprofiling analysis revealed that the combination of anti-phosphatidylserine targeting antibody and anti-PD-1 therapy enhanced tumor-infiltrating lymphocytes, and increased expression of pro-immunosurveillance-associated cytokines while significantly decreasing expression of pro-tumorigenic cytokines that were induced by single anti-PD-1 therapy.

**Conclusions:**

Our data suggest that antibody therapy targeting phosphatidylserine-associated immunosuppression, which has activity as a single agent, can significantly enhance immunotherapies targeting the PD-1 pathway in murine breast neoplasms, including triple-negative breast cancers.

## Background

In the United States alone over 200,000 women will be diagnosed with breast cancer (BC) this year, with greater than 50,000 succumbing to their disease [1]. Treatment options for those diagnosed with BC are complicated by the highly heterogeneous nature of the disease. Depending upon the subtype of BC, the majority of patients receive surgery combined with radiotherapy, hormonal therapy, or chemotherapy, and if caught early the prognosis is typically favorable [2, 3]. In addition to the use of systemic treatments, targeted therapies have succeeded in extending patient survival in some subtypes of BC, most notably those directed against the ERBB2 receptor [4–6]. Unfortunately for the treatment of triple-negative breast cancer (TNBC), one of the most aggressive subtypes of BC, no targeted therapies have shown clinical efficacy to date [7]. TNBC develops earlier in life, being frequently diagnosed in premenopausal women. TNBC patients receive surgery and chemotherapy, yet have higher relapse rates, shorter progression-free survival, and reduced overall survival than observed in other types of BC, thus underlining the need for better therapeutic treatments [8, 9].

It is now recognized that cytotoxic chemotherapeutics, such as those used to treat TNBC, can inadvertently promote an immunosuppressive tumor microenvironment through changes in the subcellular localization of the immune checkpoint regulator phosphatidylserine (PS) [10–12]. PS normally resides in the inner plasma membrane leaflet in normal mammalian cells. Environmental conditions arise in tumors that cause cellular stress, such as oxygen radicals, hypoxia, or anti-cancer treatments including irradiation and chemotherapy, causing PS in tumor-associated vascular endothelial cells and tumor cells to undergo subcellular relocalization to the outer plasma membrane with subsequent exposure to the extracellular environment [13, 14]. This relocalization in tumors promotes PS recognition and pathway activation in local immune-modulating cells that regulate both the innate and adaptive immune responses [15]. On myeloid and lymphoid cells, the TAM and TIM families of receptors recognize and bind to PS, resulting in the release of immune-suppressive cytokines, inhibition of inflammatory signaling pathways, and the attenuation of innate and adaptive cellular responses [15–19]. This may be especially pertinent for the development and expansion of CD8^+^ T cells since TIM-3, which functions as a negative regulator of TCR activation and development of a T-helper (Th) type 1 responses, binds to PS [15, 20, 21]. In addition, PS exposure in the tumor microenvironment also negatively impacts the antigen-presentation function of dendritic cells in part through downregulating MHC II function and expression [22]. Upon binding to and ingesting PS-expressing cells, dendritic cells are prevented from progressing into a mature phenotype, and are incapable of properly processing and presenting essential costimulatory molecules required for functional antigen presentation to T cells [10, 14]. Inhibition of both innate and adaptive responses by PS exposure contributes to the initiation and maintenance of an immunosuppressive microenvironment, which in turn promotes tumor progression and metastatic disease by subjugating the host’s ability to recognize and eliminate abnormal cells [23, 24].

Because of the ability of PS exposure in the tumor microenvironment to promote tumor progression, therapeutic modalities blocking the binding of PS to specific receptors on immune cells is recognized as a potential target for cancer therapy [22, 25]. In fact bavituximab, a PS-targeting antibody, is undergoing clinical evaluation for the treatment of solid tumors with encouraging results [26–28]. Studies in rodent models with PS-targeting antibodies (including mch1N11) demonstrate localization to tumor-specific PS-expressing cells and the capability to elicit an anti-tumor immune response, particularly when combined with chemotherapy or radiotherapy [12, 14, 22, 29]. While these results are encouraging, it remains to be determined in which therapeutic regimen PS-targeting antibodies will ultimately have the greatest impact on improving patient outcome: as a single agent, in conjunction with chemotherapy; or used in combination with other immunotherapies such as those targeting the checkpoint inhibitor programmed death 1 (PD-1).

The programmed death 1 (PD-1) receptor and its ligands 1 and 2 (PD-L1/L2) are key regulators in controlling the ability of tumors to downregulate the adaptive immune response and disrupt T-cell checkpoint pathways [30, 31]. PD-1 is expressed on T cells, B cells, natural killer cells, dendritic cells, and many tumor-infiltrating lymphocytes (TILs) [32]. The PD-1 ligands, PD-L1 and PD-L2, are also expressed in resting immune system cells, including T cells, B cells, and natural killer cells. In normal tissues, the PD-1:PD-L1/L2 interactions are critical inhibitory regulators that act to protect against tissue damage by limiting inflammatory reactions mediated by T cells and other immune system components during infections [32]. In cancers, tumor cell expression of PD-L1 is capable of interacting with PD-1 on cells in the tumor microenvironment, where it serves to inhibit both effector T-cell activation and cytolytic responses to tumor cells [30, 33–35]. Clinical trials using antibodies to inhibit PD-1/PD-L1 activity have shown efficacy in patients with nonsmall-cell lung cancer, melanoma, and kidney cancers [36, 37]. While further evaluation is necessary, data suggest that levels of PD-1 expression in patients’ tumors may predict response rates [38–40]. Currently, it is not known whether anti-PD-1/PD-L1 therapies will have efficacy in BCs. However, it has been demonstrated in three independent studies that upwards of 20–50 % of all BC patients, including those diagnosed with TNBC, have detectable PD-L1 levels in tumor biopsies [41–43].

Because of the ability of both PS and PD-1 to contribute to an immunosuppressive tumor microenvironment, we sought to determine whether blocking recognition of PS and PD-1 alone and in combination are effective therapies in BC models, including TNBC. Our data show that combining the PS-targeting antibody mch1N11 with anti-PD-1 antibody therapy has significantly greater inhibitory tumor growth activity and significantly increased overall survival compared with either single treatment alone, and is capable of causing complete tumor regression. In addition, mice that received combinational treatment and experienced complete tumor regression had increased splenic IFNγ production, and were capable of mounting a durable immune response that negated the growth of these TNBC cells when rechallenged. Analysis of tumors by flow cytometry showed that the combined treatment increases intratumoral TIL levels more than observed with either single treatment. Further immunoprofiling analysis demonstrated that while combinational therapy was capable of increasing the expression of pro-immune activating cell types and cytokines over that observed in single-arm treatment groups, the addition of a PS targeting antibody to anti-PD-1 therapy also has the ability to downregulate critical pro-tumorigenic cytokines, including IL-4, IL-9, and IL-17, which were induced by anti-PD-1 treatment [44–46]. These data demonstrate that antibody-mediated blockade of PS-mediated immunosuppression enhances the anti-tumor effects of PD-1 targeted therapy by further augmenting immune activation while simultaneously attenuating negative pro-tumorigenic secondary responses that accompany targeting PD-1 in TNBC.

## Methods

### Cell lines

The murine TNBC cancer line E0771 was purchased from CH3 BioSystems, LLC (Buffalo, NY, USA). EMT-6 was obtained from ATCC (Manassas, VA, USA). All cells were maintained in RPMI-1640 (GIBCO) supplements with 10 % v/v heat-inactivated fetal bovine serum (GIBCO, Waltham, MA, USA).

### Antibodies

PS-targeting antibody chimeric mch1N11 is a mouse IgG2a-kappa with human variable heavy and light chain regions; C44 is an IgG2a isotype. Purified anti-mouse PD-1 (clone RMP1-14) was obtained from BioXcell (West Lebanon, NH, USA). All in-vivo antibodies tested negative for endotoxin with a limit of detection of 0.5 EU/ml of stock antibody. Antibodies for flow cytometer analysis were CD45 (clone 30-F11), CD3e (clone 17A2), CD4 (clone GK1.5), CD8a (clone 53–6.7), and CD25 (clone PC61.5), all obtained from Bioscience (San Diego, CA, USA). Antibodies for immunohistochemistry were anti-PD-L1 antibody (clone MIH6, catalog number ab80276; Abcam, Cambridge, MA, USA) and anti-PD-1 antibody (clone RMP1-14, catalog number ab63477; Abcam).

### Immunohistochemistry

E0771 and EMT-6 tumors were imbedded in OCT medium and frozen in a dry-ice isopentane bath. Tissue sections were cut (5 μm) and stained for PD-L1 and PD-1 positive cells. Tissue samples were stained with secondary antibodies labeled with peroxidase or alkaline phosphatase followed by counterstain with hematoxylin and analyzed by bright-field microscopy. Slides were digitized using a Lumenera infinity digital camera and version 6.4 software (Lumenera, ON, Canada).

### Animals

Female C57BL/6 or Balb/c mice 4–6 weeks old were purchased from Charles River Laboratories (Wilmington, MA, USA). All studies were approved by the Institutional Animal Care and Use Committee at the University of California, Irvine, USA.

### In-vivo studies

EMT-6/E0771 tumor cells were suspended at 1 × 10^7^/ml in Matrigel (50 % v/v) in PBS and 0.1 ml was injected into the 9/10 mammary fat pad (E0771, C57BL/6; EMT-6, Balb/c). Tumor volumes (*V*) were calculated using the formula:

$$ V=\left(L\times W\times H\right)\times 0.5 $$

where *L* is the length, W is the width, and *H* is the height of the tumor. The percent tumor growth inhibition (% TGI) was calculated using the formula:

$$ \%\ \mathrm{T}\mathrm{G}\mathrm{I}=1\ \hbox{--} \left(\mathrm{T}/\mathrm{C}\right)\times 100 $$

where *T* is the mean tumor volume of the treated group at the end of study and *C* is the mean tumor volume of the control group at the end of study. For tumor rechallenge studies, animals with no palpable tumor were injected with E0771 cells under the same initial dosing conditions but on the opposing mammary fat pad (4/5). The tumor rechallenge response endpoint was expressed as tumor growth delay and the difference in time (days) was calculated between the growth delay of the treated group and the naïve control group. All treatment was administered via intraperitoneal injection in 100 μl volumes twice weekly (C44 control, 10 mpk; mch1N11, 10 mpk; anti-PD-1 2.5 mpk; and mch1N11 + anti-PD-1, 10/2.5 mpk respectively). Doses were selected though preliminary maximum tolerated dose (MTD) studies (data not presented), and no toxicity/weight loss was encountered in the data presented.

### IFNγ EliSpot

Spleens were obtained from naïve nontumor-bearing mice that were untreated, single, or combination treated, or from E0771 tumor-bearing mice treated with C44, or from animals with regressed E0771 tumors following treatment with mch1N11 and anti-PD-1. Spleens were harvested on day 12 following tumor implantation or from nontumor animals following a matching treatment regimen. Single-cell preparations of splenocytes were resuspended in RPM1-1640 supplemented with 10 % FCS containing antibiotics at 1 × 10^6^ cells/ml and 100 μl added, in triplicate, to wells of EliSpot microplates coated with anti-mouse IFNγ IgG, in the absence or presence of 1 × 10^5^ irradiated (15,000 rad) E0771 cells to determine tumor-specific stimulation. Plates were incubated for 48 h at 37 °C and spots were developed using anti-mouse IFNγ IgG–HRP conjugate followed by peroxidase substrate. Spots were counted using an automated EliSpot plate reader.

### Flow cytometry

Tumors were excised from mice and physically dissociated and digested in 1 mg/ml collagenase (Sigma, St. Louis, MO, USA), 0.1 mg/ml hyaluronidase (Sigma, St. Louis, MO, USA), and 200 units/ml DNase type IV (Sigma, St. Louis, MO, USA) for 1.5 h at 37 °C and passed through a 70 μm sieve filter (Falcon, Corning, NY, USA). Cells were collected, treated with ACK lysis buffer to remove red blood cells, washed twice with PBS, resuspended in FACS staining buffer, and stained with antibodies for 20 min at 4 °C.

### NanoString immunoprofiling analysis

E0771 RNA was prepared from six tumors for each treatment group shown in Fig. 2a at study end (day 26) by Direct-zol™ RNA mini prep kit (ZymoResearch, Irvine, CA, USA). Gene expression was directly measured via counts of corresponding mRNA in each sample using an nCounter (NanoString, Seattle, WA, USA) GX murine PanCancer Immune Profiling Panel, which is a multiplex assay for 770 genes involved in the murine inflammatory response [47]. The nCounter system allows for direct detection and counting of nucleic acid via reporter probes appended with multiple fluorophore barcodes and biotinylated capture probes that attach to microscopic beads, which are then affixed to lanes in a translucent cartridge and read in an optical scanner. Batches of 12 separate samples (six from each treatment group) at one time were prepared as per the manufacturer’s instructions, with 100–300 ng of total RNA hybridized with probes at 65 °C for 16–18 h before being placed into the automated nCounter Prep in which samples were affixed to cartridges. Cartridges were then immediately placed into the nCounter Digital Analyzer optical scanner and read at a goal resolution of 550 fields of view, which is the maximum resolution for this instrument. Analysis was performed using the nSolver 2.6 analysis software with immune cell types based upon classifications by Newman et al. and Bindea et al. [48, 49]. Probes used to classify cell types were as follows; T cells (CD2, CD3e, CD3g, CD6g), regulatory T cells (Tregs) (FoxP3), macrophages (CD68,CD163,CD84), Th1 cells (CD38, Ctla4, IFN-g, LTA, Stat4, Tbx1), Th2 cells (Cxcr6, Birc5, Gata3 Pmch, Stat6), and dendritic cells (CCl17, Cdl7, Ccl22, Cd1d2).

### Statistical analysis

Statistical analysis was performed using Student’s *t* test and expressed as median with SD, with the exception of analysis for Kaplan–Meier survival curves which utilized Mantel–Cox analysis. *p* < 0.05 was considered significant.

## Results

### Expression of PD-1 and PD-L1 in E0771 and EMT-6 BC cells and tumors

**Fig. 1:**
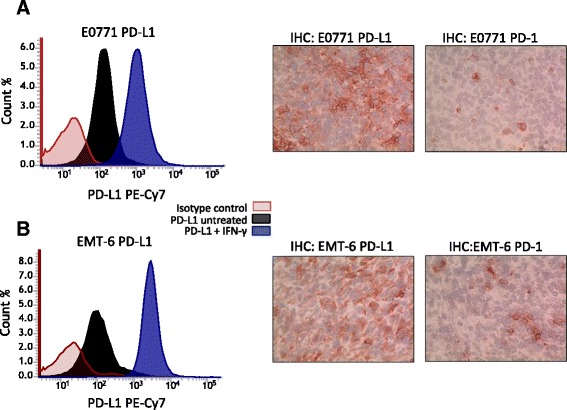
Expression of PD-L1 and PD-1 in breast tumors. FACS and immunohistochemistry (*IHC*) analysis of **a** E0771 and **b** EMT-6 cells grown in vitro to determine whether PD-L1 is constitutively expressed and/or is inducible by IFNγ treatment. *PD-1*, programmed death 1, *PD-L1* programmed death-1 ligand 1

In-vitro PD-L1 expression on E0771 and EMT-6 cells was examined by flow cytometry. As shown in Fig. 1a, each BC cell line had basal PD-L1 expression, with E0771 cells having slighter higher levels. When cells were stimulated with IFNγ, levels of PD-L1 increased substantially in each cell line compared with untreated cells, with levels in EMT-6 cells reaching higher expression levels than those observed in E0771 cells. Tumors derived from implanted E0771 and EMT-6 cells also had detectable expression of PD-1 and PD-L1. As shown in Fig. 1b, PD-1 and PD-L1 were detectable in both E0771 and EMT-6 tumor samples by immunohistochemistry, thus supporting the evaluation of combination therapy by antibodies targeting PS and the PD-1/PD-L1 axis in these murine models.

### Effect of PS targeting and PD-1 inhibition on in-vivo growth in E0771 and EMT-6 cells

**Fig. 2:**
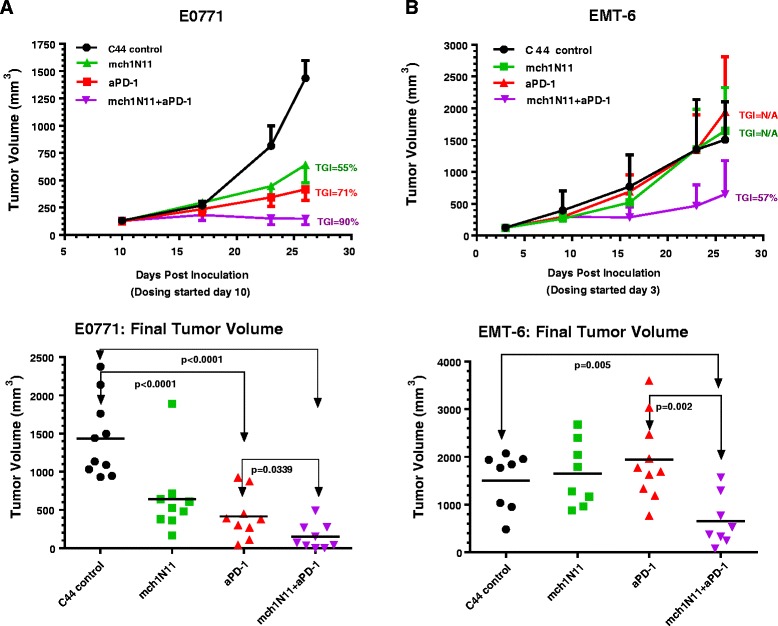
Anti-tumor effects of PS and PD-1 targeting alone and in combination in murine BC models. **a** *Top panel*: growth kinetics of E0771 TNBC tumors in C57/Bl6 mice treated with control (C44 ), mch1N11, anti-PD-1, or mch1N11 + anti-PD-1 antibody combinations. Treatments started at 10 days post inoculation when tumors were approximately 100 mm^3^. All data points are expressed as median and SD. *Bottom panel*: analysis of E0771 final tumor volumes from the TGI study in *upper panel*. Statistical analysis (Student’s *t* test) demonstrates that combinational treatment with PS-targeting antibody and ant-PD-1 antibody has significant inhibitory effects compared with all other treatments. **b** *Top panel*: growth kinetics of EMT-6 murine breast tumors in Balb/c mice treated with control (C44), mch1N11, anti-PD-1, or mch1N11 + anti-PD-1 antibody combinations. Treatments started at 3 days post inoculation when tumors were approximately 100 mm^3^. All data points are expressed as median and SD. *Bottom panel*: analysis of EMT-6 final tumor volumes from TGI study in *upper panel*. Statistical analysis demonstrates that combinational treatment with PS-targeting antibody and anti-PD-1 antibody has significant inhibitory effects compared with all other treatments. Statistically significant differences between groups were identified by Student’s *t* test, with 10 animals in each group. *TGI* tumor growth inhibition

To determine the therapeutic effect of PS and PD-1 targeting alone and in combination, mice were implanted orthotopically with cells from the murine TNBC line E0771. When tumors reached approximately 100 mm^3^ (day 10), treatments commenced with twice-weekly dosing for a total of six doses (3 weeks) by intraperitoneal injection. Treatment by the PS-targeting antibody, mch1N11 (10 mpk), resulted in 55 % TGI compared with control treatment (C44) (Fig. 2a, upper panel). Treatment by anti-PD-1 antibody (2.5 mpk) resulted in a TGI of 71 % compared with control treatment. Combinational treatment of mch1N11 (10 mpk) with anti-PD-1 (2.5 mpk) antibody significantly suppressed E0771 growth by 90 % compared with control (Fig. 2a, upper panel). Comparison of the final tumor volumes in each treatment group demonstrated that, in addition to significantly suppressing the growth of E0771 compared with control treatment (*p* < 0.0001), combination treatment by mch1N11and anti-PD-1 significantly inhibited tumor growth more than treatment by either single arm alone (Fig. 2a, lower panel, *p* = 0.0339 to anti-PD-1 treatment). In the EMT-6 BC model, unlike the results observed in the E0771 model, mch1N11 (10 mpk) and the anti-PD-1 blocking antibody (2.5 mpk) had no growth inhibitory effects as single agents (Fig. 2b, upper panel). However, similar to the results from combination treatment in the E0771 model, combining PS-targeting and anti-PD-1 therapies together was capable of significantly impeding tumor growth in the EMT-6 model (TGI = 57 %, *p* = 0.005 to control treatment, Fig. 2b upper and lower panels). No loss of weight was noted in any of the treatment groups in the E0771 or EMT-6 models (data not shown).

### Effect of anti-PD1 and PS-targeting antibodies on overall survival in a murine BC model

**Fig. 3:**
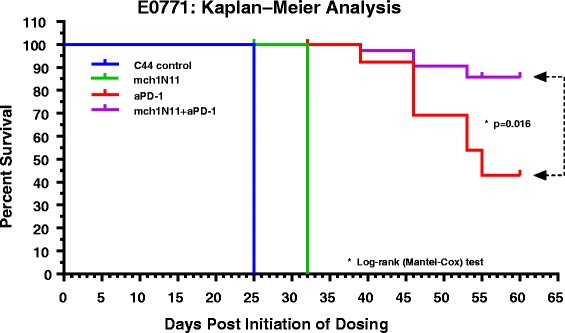
Effect of PS-targeting and anti-PD-1 antibody therapy on survival in a TNBC murine model. Mice with E0771 tumors were treated a total of six times once tumors reached approximately 100 mm^3^ (days 1, 4, 8, 11, 15, and 18) with single antibody or a combination of mch1N11 and anti-PD-1, and survival times were determined. Once tumors reached approximately 1500 mm^3^ or animals encountered a tumor-related health problem, animals were euthanized. Survival times were plotted and determined by Kaplan–Meier analysis, statistical analysis was determined by log rank (Mantel–Cox) analysis

To determine whether treatment by mch1N11 and anti-PD-1 therapy prolonged overall survival in a BC model, mice inoculated with E0771 tumors were treated with control antibody (C44, 10 mpk), mch1N11 (10 mpk), anti-PD-1 antibody (10 mpk), or the combination of mch1N11 plus anti-PD-1 antibody (10 mpk/10 mpk). As shown in Fig. 3 and summarized in Table 1, all control mice had tumors that progressed with treatment by the C44 isotype antibody and survived until day 25 of treatment, when the end point was reached due to excessive tumor burden. All mice receiving mch1N11 antibody also progressed while receiving treatment, but were able to survive for an additional 7 days beyond control mice. Mice receiving anti-PD-1 therapy alone at 10 mpk survived significantly longer than those receiving control or mch1N11 treatments, having a median survival time of 55 days. Mice receiving the combination of mch1N11 with anti-PD-1 antibody experienced a significant increase (*p* = 0.0155) in overall survival compared with single anti-PD-1 treatment, with six of 10 mice having complete tumor regression by day 25, compared with two of 10 mice in the anti-PD-1 group, accompanied by no tumor reoccurrence 30 days after the study end (day 60) in all animals with complete regression (Table 1).

**Table 1:** Summary of Kaplan–Meier survival analysis of the E0771 in-vivo study (Fig. 3)

	Median survival			
	Control (c44)	mch1N11	aPD-1	mch1N11 + aPD-1
Days	25	32	55	Undefined
Hazard ratio	–	0.1915	0.089	0.04557
CI of HR at 95 %	–	0.003086–0.03911	0.0001420–0.004134	3.222 × 10^–5^–0.001361
Mice with complete regression	0	0	2	6

Data include median survival (days), hazard ratio (HR) confidence interval (CI) at 95 % of HR, and number of animals with each treatment that experienced complete tumor regression

### Effect of PD-1 and PS treatments on developing and maintaining a durable anti-tumor response to secondary tumor challenge by TNBC cells

**Fig. 4:**
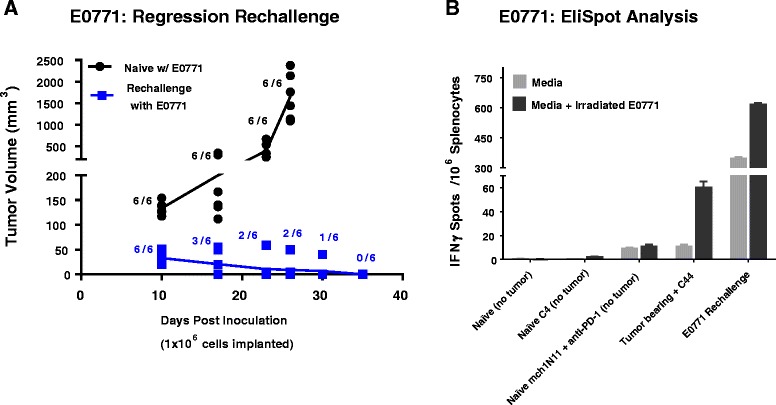
Effect of mch1N11 and anti-PD-1 therapy on establishing an immune-mediated resistance to E0771 rechallenge and presence of functional T cells. **a** Animals that received mch1N11 + anti-PD-1 treatment and underwent a complete tumor regression (Fig. 4) were rechallenged, along with naïve C57BL/6 mice, with E0771 cells (1.0 × 10^6^). Cells were inoculated in the opposing mammary fat pad (4/5) from the previous initial inoculation (8/9). The presence of palpable tumors and growth kinetics was determined for each group. **b** EliSpot analysis of IFNγ in splenocytes cultured with and without irradiated E0771 cells. Splenocytes from nontumor-bearing control animals (no treatment, C44, and mch1N11 + anti-PD-1 treated), tumor-bearing animals (C44 treated), and mice resistance to E0771 rechallenge (Fig. 4a, 4b) were utilized. All analyses were performed in triplicate

To elucidate whether mice that had complete tumor regression in the mch1N11 plus anti-PD-1 treatment group (Fig. 3 and Table 1) were resistant to E0771 rechallenge, the six mice that experienced complete tumor regression and six control naïve mice were inoculated with E0771 cells in the 4/5 mammary fat pad and tumor growth was measured. After 10 days, the naïve mice had an average tumor volume of 134 ± 5.3 mm^3^ compared with 32.8 ± 4.6 mm^3^ in the E0771 rechallenged group (Fig. 4a). On day 17, only three of the six mice in the rechallenged group had detectable tumors with an average volume of 20.8 ± 10.5 mm^3^, compared with 199.8 ± 39.5 mm^3^ in control mice. On day 26 all mice in the control group reached maximum study tumor volume limits (1656 ± 210 mm^3^) while the tumors continued to regress in the rechallenged group, with only two mice having detectable tumors (average 9.2 ± 8.2 mm^3^). By day 35 no tumors were detected in the rechallenge group (Fig. 4a), and no tumor growth was noted for an additional 14 days (data not shown).

### Effect of PS-targeting and anti-PD-1 antibody treatment on splenocyte IFNγ production

To ascertain whether the mice that experienced complete tumor regression with mch1N11 and anti-PD-1 treatment and were subsequently resistant to E0771 rechallenge (Fig. 4a) had an enhanced tumor-specific immune response, we evaluated IFNγ production in splenocytes cultured with and without irradiated E0771 cells by EliSpot (Fig. 4b). Splenocytes from nontumor-bearing control animals (no treatment and C44 treated) had similar IFNγ production when cultured in media alone (naïve, 0.40 ± 0.23; naïve + C44 treated, 0.27 ± 0.27). When cultured in the presence of irradiated E0771, there was a slight increase in IFNγ production in naïve mice treated with the C44 control antibody compared with nontreated mice (naïve, 0.13 ± 0.13; naïve + C44 treated, 2.0 ± 0.46). In nontumor-bearing mice that received mch1N11 and anti-PD-1 treatment, there was a slight increase in IFNγ production when cultured in media alone (9.0 ± 0.81); however, this signal did not increase when irradiated E0771 cells were cocultured with splenocytes from combination-treated animals (10.9 ± 1.71). Splenocytes from tumor-bearing mice that received C44 control antibody had a signal of 10.7 ± 1.7 when grown in media alone, and IFNγ production increased to 60.1 ± 4.8 spots per well with coculturing with irradiated E0771 cells. In the animals with regressed tumors following mch1N11 and anti-PD-1 treatment, and further resistance to E0771 rechallenge (Fig. 4a, b), IFNγ EliSpots were significantly higher than observed under previous treatments and conditions. Splenocytes from these animals cultured in media alone had a baseline signal of 347 ± 7.9 spots per well, suggesting mch1N11 and anti-PD-1 treatment increases the number of functional T cells in the spleens of combination-treated animals, perhaps leading to the observed tumor regression and subsequent rejection of tumor recolonization with E0771 reinoculation. The addition of irradiated E0771 cells to these splenocytes elevated the signal to 614.7 ± 9.7 spots per well, demonstrating that E0771 antigens are capable of further stimulating IFNγ production in T cells from mch1N11 and anti-PD-1-treated animals (Fig. 4b).

### Effect of PS-targeting and anti-PD-1 antibodies on TNBC TILs

**Fig. 5:**
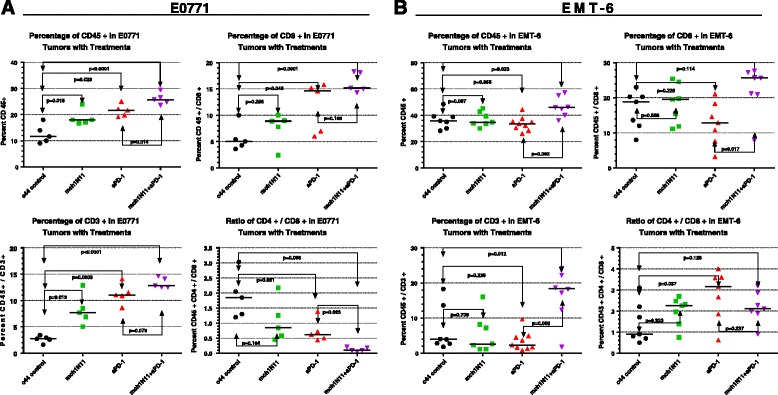
Effect of PS-targeting and anti-PD-1 therapies on TILs in E0771 and EMT-6 tumors. **a** Mice with E0771 tumors were treated on days 10, 14, 17, 21, and 25 post implantation with single antibody treatments or a combination of mch1N11 and anti-PD-1. Five tumors from each treatment group were excised on day 26 and single-cell preparations were stained with antibodies specific to CD45^+^, CD8^+^, CD4^+^, and CD3^+^. Data are expressed as the group median and percentages of an individual animal positive for a specific surface marker by FACS analysis. CD8^+^, CD3^+^, and CD4^+^ cells were all subpopulations of CD45^+^ cells. **b** Mice with EMT-6 tumors were treated on days 10, 14, 17, 21, and 25 post implantation with single antibody treatments or a combination of mch1N11 and anti-PD-1. Five tumors from each treatment group were excised on day 26 and single-cell preparations were stained with antibodies specific to CD45^+^, CD8^+^, CD4^+^, and CD3^+^. Data are expressed as the group median and percentages of individual animal positive for a specific for a surface marker by FACS analysis. CD8^+^, CD3^+^, and CD4^+^ cells were all subpopulations of CD45^+^ cells

The anti-tumor response induced by mch1N11 in combination with anti-PD-1 in mice bearing the TNBC model E0771 tumors suggests that treatment by PS and PD-1-targeting antibodies may induce TIL changes within the tumor microenvironment. Examination of excised E0771 tumors at the study end in Fig. 2a demonstrated that treatment by mch1N11 increased the presence of CD45^+^ cells from a median value of 11.6 % in control (C44) tumors to 18 % (*p* = 0.0179 to control) with PS targeting, while anti-PD-1 therapy increased CD45^+^ levels to 21 % (Fig. 5a). In combination treated mice, the addition of mch1N11 to anti-PD-1 therapy significantly increased CD45^+^ levels to 25.7 % (*p* < 0.0001 to C44 control, *p* = 0.0139 to anti-PD-1 single treatment). Examination of CD8^+^ and CD3^+^ levels (CD8^+^, CD3^+^, and CD4^+^ cells were all subpopulations of CD45^+^ cells) showed a similar trend in response to treatments. Single mch1N11 treatments increased CD8^+^/CD3^+^ levels to 8.9 %/7.7 % respectively over control levels (CD8^+^ = 5.7 %, CD3^+^ = 2.7 %). Single anti-PD-1 therapy increased CD8^+^ and CD3^+^ levels to 14.6 % and 11 % respectively, while the combination of PS with anti-PD-1 therapy elevated CD8^+^ levels to 15.2 % and CD3^+^ levels to 12.8 %. Examination of the CD4^+^ to CD8^+^ T-cell ratio demonstrated that while each single arm treatment group decreased the percentage of CD4^+^ to CD8^+^ cells, combinational therapy further significantly decreased this ratio (*p* = 0.023), suggesting combined treatment was capable of further reducing the CD4 T-cell helper populations and increasing the effector CD8^+^ cells in the tumor microenvironment (Fig. 5a). FACS examination of the EMT-6 in-vivo study (Fig. 2b) demonstrated that, unlike our observations in the E0771 model, single-arm treatments had little observable effect on altering TILs (Fig. 5b). C44 control CD45^+^ levels had a median value of 36.8 %, while mch1N11 had a value of 34.7 % and anti-PD-1 of 33.4 %. Only in the combination group was an increase in CD45^+^ noted (46.0 %, *p* =0.023). In CD8^+^ populations, C44 control tumors were 12.0 %, with mch1N11 of 11.80 % and anti-PD-1 exhibiting 7.6 %. The combination treatment group did increase to 20.9 %, but the results were not significant (*p* = 0.112). Similar results occurred in CD3^+^ populations. C44 control contained 3.9 % CD3^+^ cells, while mch1N11 showed 2.6 % and anti-PD-1 treatment showed 2.3 %. Again only the combinational treatment arm showed a significant increase to 18.4 % (*p* = 0.012, Fig. 5b). Examination of the ratio of CD4^+^ to CD8^+^ T cells in EMT-6 tumors demonstrated that all of the treatment arms slightly increased the percentage of CD4 cells to CD8 cells; however, none of the changes were statistically significant for control treatment (C44 control, 0.9 %; mch1N11, 2.3 %; anti-PD-1 3.2 %; and mch1N11 + anti-PD1, 2.1 %).

Combined, our results in the E0771 model demonstrate that while each single arm treatment is capable of increasing CD45^+^, CD8^+^, and CD3^+^ populations in E0771 tumors, combining PS-targeting and anti-PD-1 antibodies increases the percentage of TILs over that observed with single treatments. Contrary to these results, only the combination of the PS blocking antibody treatment with anti-PD-1 treatment in the EMT-6 model was capable of inducing TILs, reflecting the TGI data in Fig. 2a, b.

### Effect of PS-targeting and anti-PD-1 therapy on RNA immunoprofile in TNBC tumors

**Fig. 6:**
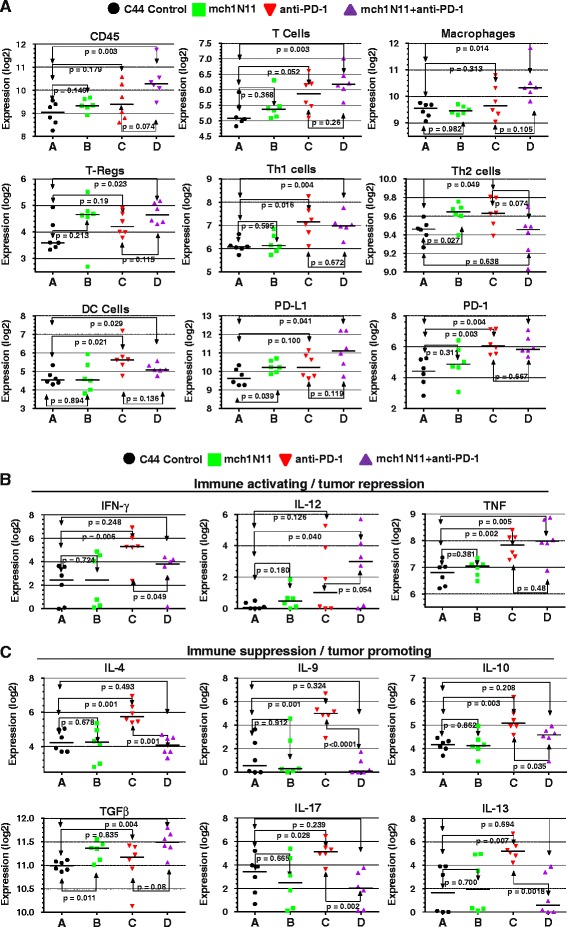
NanoString immune-profiling analysis of tumor samples from control (C44), PS (mch1N11), and anti-PD-1 therapy. RNA isolated from tumors obtained at the study termination (shown in Fig. 3) was subjected to analysis utilizing the NanoString™ nCounter PanCancer Immune Profiling Panel. Expression values, expressed in log_2_ are graphically represented. **a** Profiling of tumor-associated immune cell type markers, including the markers for PD-1 and its ligand PD-L1. **b** Expression profiling of immune-activating-associated cytokines in each treatment group. **c** Expression profiling of immune-suppression-associated cytokines in each treatment group. *DC* dendritic cell, *PD-1* programmed death 1, *PD-L1* programmed death-1 ligand 1, *Th* T-helper, *Treg* regulatory T cell

To further capture and understand changes in the immune landscape that accompany PS-targeting and anti-PD-1 therapies, RNA isolated from tumors obtained at the study termination (shown in Fig. 2a) were subjected to mRNA expression analysis utilizing NanoString™ PanCancer Immune Profiling Panel analysis based on classifications described by Newman et al. and Bindea et al. [48, 49]. Differences in the expression patterns of genes associated with CD45^+^, T cells (probes; CD2, CD3e, CD3g, CD6g), and tumor-associated macrophages (probes; CD68, CD163, CD84) showed that while single treatments were capable of increasing the levels of these cell types, most notable for CD45^+^ and T cells, over control (C44) levels, combination treatment further enhanced levels of these cell types compared with each single treatment (Fig. 6a). Increased Treg (probe; Foxp3) levels occurred in all treatment groups compared with the control, with anti-PD-1 therapy having slightly less overall levels compared with treatments that included a PS-blocking antibody. Examination of Th1 (probes; CD38, Ctla4, IFN-g, LTA, Stat4, Tbx1) and Th2 (probes; Cxcr6, Birc5, Gata3 Pmch, Stat6) immunoprofiles showed that distinct changes occurred with each type of treatment. Th1 levels were increased only when PD-1 therapy was included, either as a single treatment or in combination with PS-blocking antibodies, while Th2 levels increased over control levels with each single treatment, and combination therapy brought Th2 levels down to those observed in control treatment mice (Fig. 6a). Examination of dendritic cell markers (probes; CCl17, Cdl7, Ccl22, Cd1d2) demonstrated that PS treatment did not increase levels over control, while anti-PD-1 alone and when combined with mch1N11 both increased levels similarly, suggesting that anti-PD-1 treatment increased dendritic cell levels. Finally we examined levels of PD-L1 and PD-1. Both single-arm treatments similarly increased PD-L1 levels; however, combination treatment increased PD-L1 mRNA levels over that observed in each single arm (Fig. 6a). PD-1 levels increased slightly with mch1N11 compared with control, while treatment with anti-PD-1 alone or in combination similarly induced PD-1 mRNA levels in E0771 tumors. These data suggest that combinational treatment with PS-targeting and anti-PD-1 antibodies increase immune cell infiltration (CD45, T cells, macrophages) over each single-arm treatment and that treatment with PS and anti-PD-1 is capable of inducing PD-L1 levels which may further serve as available targets for intervention with anti-PD-L1/PD-1 therapeutics.

### Effect of PS-targeting and anti-PD-1 antibody therapy on expression of pro-oncogenic and anti-oncogenic associated cytokines in the TNBC

Numerous secreted cytokines and immunomodulatory proteins are expressed in the tumor microenvironment that are associated with immune system activation or suppression [50, 51]. We examined the levels of cytokine IFNγ, IL-10, and TNF mRNA in tumors following treatments with single or a combination of mch1N11 and anti-PD-1 antibody. IFNγ levels did not change with mch1N11; however, anti-PD-1 single treatment and mch1N11 and anti-PD-1 antibody combination increased IFNγ levels, with the anti-PD-1-alone group having the highest levels (Fig. 6b). Levels of IL-12 and TNF increased compared with control in both single-arm treatment groups, with combination treatment further enhancing expression of each of these immune promoting factors over that observed in either single treatment arm (Fig. 6b). Further examination of immunosuppression associated cytokines, including IL-4, IL-9, IL-10, IL-13, IL-17, and TGFβ, revealed the complex changes associated within each of our treatment groups (Fig. 6c). Levels of IL-4 and IL-9 were unchanged from control levels in the mch1N11 and combinational mch1N11 and anti-PD-1 treatment groups; however, anti-PD-1 treatment alone dramatically induced IL-4 and IL-9 levels (Fig. 6c). IL-10 levels were also unaffected by mch1N11 as a single agent; however, anti-PD-1 therapy alone increased levels the most, with the addition of PS antibody to anti-PD-1 therapy reducing IL-10 levels. Similar induction of the immunosuppressing/tumor-promoting cytokines IL-17 and IL-13 was noted with anti-PD-1 treatment, with combination treatment reducing levels below those observed in C44 control mice. The only immunosuppressive/pro-oncogenic cytokine assayed that showed an increase in mice receiving mch1N11 therapy over that observed in the anti-PD-1 single treatment arm was TGFβ. While all treatments increased TGFβ levels over those observed in control tumors, the combination of PS-targeting and anti-PD-1 antibodies induced levels higher than observed in each single treatment arm (Fig. 6c).

## Discussion

TNBC represents one of the most malignant subtypes of BC. Current treatment strategies for patients diagnosed with TNBC rely on systemic cytotoxic chemotherapeutics, causing exposure of PS in the tumor microenvironment, which in turn promotes an immunosuppressive condition [14, 52]. Previous work has demonstrated that PS-driven immune suppression can be blocked or reversed by PS-targeting antibodies [14, 22]. In this study, we sought to ascertain whether inhibition of PS-mediated immune suppression is capable of enhancing, or acting synergistically, when combined with an inhibitory treatment targeting the downstream checkpoint regulator PD-1 in murine BC models, including TNBC. Here we demonstrate that PS-targeting antibodies combined with anti-PD-1 therapy have significantly greater anti-TGI than either treatment alone (Fig. 3a, b). In the TNBC E0771 model, each single therapy had moderate activity (mch1N11, TGI = 55 %; anti-PD-1, TGI = 71 %), but combination treatment was capable of achieving significant TGI over either single therapy (TGI = 90 %, *p* = 0.00339 compared with anti-PD-1). Interestingly, in the EMT-6 breast model, single treatments by PS-targeting or anti-PD-1 antibodies had no discernible anti-tumorigenic effect and did not modulate TILs. However, combining PS therapy with anti-PD-1 therapy in the EMT-6 model showed activity and achieved a TGI of 57 % and significantly increased CD45^+^ and CD3^+^ levels. While it is unclear why single-arm treatments did not increase TILs, the higher baseline of CD45^+^ cells in the EMT-6 model (46.0 %) compared with the E0771 model (11.6 %) may suggest a higher percentage of myloid derived supressor cells (MDSC) or Tregs in the EMT-6 tumor microenvironment that single-arm treatments are unable to overcome. Regardless, our data in the EMT-6 model suggest that combining PS-targeting antibodies with PD-1-targeting therapies in BCs may have activity regardless of sensitivity to single treatments, although this requires further validation and investigation into the potential mechanism(s) that contribute to this sensitivity.

Similar to our studies examining the anti-tumor growth effects of PS-targeting antibodies with and without anti-PD-1 therapy, we also demonstrate that combinational therapy provides a distinct advantage over single-arm treatments in the TNBC E0771 model (Fig. 3). PS-targeting antibodies alone provided an additional 7 days of survival over that in the control (32 vs. 25 days), while anti-PD-1 therapy increased the survival time to an average of 55 days. Combination treatment of PS-targeting with anti-PD-1 antibodies significantly extended the survival time (*p* = 0.0155 to single anti-PD-1 treatment), with the average survival time being undefined due to six of the 10 animals experiencing complete tumor regression compared with two of 10 animals in the anti-PD-1 group (Fig. 3).

In addition to demonstrating that combination treatment inhibits tumor growth and extends overall survival, we also show that PS-targeting antibodies and anti-PD-1 treatment is capable of activating immune system recognition and elimination of secondary tumor challenge by TNBC cells. In the animals that experienced complete tumor regression with combinational treatment, tumor cell reinoculation in the adjacent mammary fat pad failed to establish any tumors and tumor cells were rapidly eliminated compared with naïve animals inoculated simultaneously with an equal number of E0771 cells (Fig. 4a). Animals that experienced the ability to reject secondary challenge also showed elevated IFNγ production in their splenocytes compared with naïve animals treated with either control or PS-targeting and anti-PD-1 therapy, which was further elevated by stimulation with irradiated E0771 cells (Fig. 4b). Furthermore, while tumor-bearing animals treated with control antibody did have elevated IFNγ levels compared with nontumor-bearing animals, the levels of IFNγ were 20-fold lower than levels observed in animals that had tumor regressions following combination treatment (Fig. 4b). IFNγ has multiple immunoregulatory effects on a variety of cell types, including the capacity to promote the activation of cytotoxic T lymphocytes, natural killer cells, and macrophages, in addition to inducing and enhancing expression of class II MHC antigens [53, 54]. Studies demonstrate that while IFNγ is essential for the development and ability of T cells to mount a specific anti-tumor response, it is also essential for the ability of T cells generated in lymphoid organs, including the spleen, to migrate to tumor sites and mount an anti-tumor response [55, 56].

The importance and prognostic value of TILs in BC is controversial [57]. BC has been generally considered a cancer not amenable to immunotherapeutic interventions. However, emerging studies have suggested that in TNBC patients the presence of TILs may be an indicator of higher response rates to neoadjuvant chemotherapy and reduced distant reoccurrence, and may predict better overall survival [58–60]. Our studies demonstrate that combining PS-targeting antibody and anti-PD-1 therapies increases levels of TILs more than observed by either treatment as a single agent, and this increase correlates with greater anti-tumor growth effects and better overall survival. Examination of chemokines in the E0771 model associated with enhancing TILs (including Ccl3 and Ccl4) showed a general trend of increased expression in the combination groups over each single arm; however, the results were not statistically significant (data not shown). We also observed increased levels of tumor-associated macrophages with combination treatment by NanoString analysis. While we were are unable to distinguish whether a M1/M2 repolarization event occurred in the macrophage population with the murine NanoString analysis, the increase in TNF and IL-12 accompanied by a decrease in IL-10 levels suggest that a more M1-like phenotype may occur with combinational therapy. A shift to a M1 phenotype using a PS blocking antibody has been reported previously [22], but the prognostic value of the increase in the overall percentage of macrophages remains unknown [61–63].

Continued examination of our tumor immunoprofiling of soluble factors demonstrates that with the exception of IFNγ, which was highest in the anti-PD-1 treatment group, combination treatment increased levels of the immune-surveillance maturation and promoting cytokines IL-12 and TNF over all other treatment groups, suggesting that while PD-1 treatment is capable of inducing these anti-tumor factors, including PS-targeting antibodies with anti-PD-1 treatment is capable of further stimulating their production. In addition, we observed elevated cytokine expression levels of IL-4, IL-9, IL-10, IL-13, and IL-17 in the single anti-PD-1 treatment group, which were reduced in tumors from animals treated with PS-targeting and anti-PD-1 treatments to levels equal to or less than observed in the control group (Fig. 6c). These cytokines are linked to both immunosuppressive and tumor-promoting conditions in BCs. IL-4 is associated with enhanced metabolic pathways in BC, thereby promoting tumor cell growth and metastatic disease [45]. IL-9 impedes adaptive immunity responses and the maturation of T cells, while IL-10 can assist in tumor immune surveillance escape [46, 64]. IL-13 and IL-17 expression are associated with the promotion of metastatic disease in BCs, including those that are negative for estrogen receptor expression [65, 66]. This suggests that PS-targeting antibodies may further enhance the pro-inflammatory/anti-tumor cytokine profile in the tumor microenvironment by decreasing immunosuppressive/pro-tumorigenic cytokine expression induced by anti-PD-1 therapy.

## Conclusions

In summary, our observations demonstrate that including PS-targeting antibodies such as bavituximab can enhance the anti-tumor activity of anti-PD-1/PD-L1 treatments, not only by increasing TIL responses but also by inhibiting cytokines stimulated by single-agent anti-PD-1 therapy that serve to suppress the immune response and promote tumor progression.

## Abbreviations

BC: breast cancer
PD-1: programmed death 1
PD-L1: programmed death-1 ligand 1
PD-L2: programmed death-1 ligand 2
PS: phosphatidylserine
TGI: tumor growth inhibition
Th: T-helper
TIL: tumor-infiltrating lymphocyte
TNBC: triple-negative breast cancer
Treg: regulatory T cell
